# Dengue Virus and Zika Virus Seroprevalence in the South Pacific Populations of the Cook Islands and Vanuatu

**DOI:** 10.3390/v16050807

**Published:** 2024-05-19

**Authors:** Charlotte E. B. Saretzki, Gerhard Dobler, Elizabeth Iro, Nicole Heussen, Thomas Küpper

**Affiliations:** 1 Institute for Occupational, Social and Environmental Medicine, RWTH Aachen Technical University, 52074 Aachen, Germany; tkuepper@ukaachen.de; 2 Bundeswehr Institute of Microbiology, 80937 Munich, Germany; gerharddobler@bundeswehr.org; 3 Cook Islands Ministry of Health, Rarotonga P.O. Box 109, Cook Islands; elizabeth.iro@cookislands.gov.ck; 4 Department of Medical Statistics, RWTH Aachen Technical University, 52074 Aachen, Germany; nheussen@ukaachen.de; 5 Faculty of Medicine, Sigmund Freud University, 1020 Vienna, Austria; 6 Faculty for Travel Medicine, Royal College of Physicians and Surgeons of Glasgow, Glasgow G2 5RJ, UK

**Keywords:** dengue, Zika, chikungunya, arboviruses, seroprevalence, South Pacific, island populations

## Abstract

Arboviral diseases are serious threats to global health with increasing prevalence and potentially severe complications. Significant arthropod-borne viruses are the dengue viruses (DENV 1-4), the Zika virus (ZIKV), and the chikungunya virus (CHIKV). Among the areas most affected is the South Pacific Region (SPR). Here, arboviruses not only cause a high local burden of disease, but the region has also proven to contribute to their global spread. Outpatient serum samples collected between 08/2016 and 04/2017 on three islands of the island states of Vanuatu and the Cook Islands were tested for anti-DENV- and anti-ZIKV-specific antibodies (IgG) using enzyme-linked immunosorbent assays (ELISA). ELISA test results showed 89% of all test sera from the Cook Islands and 85% of the Vanuatu samples to be positive for anti-DENV-specific antibodies. Anti-ZIKV antibodies were identified in 66% and 52%, respectively, of the test populations. Statistically significant differences in standardized immunity levels were found only at the intranational level. Our results show that in both the Cook Islands and Vanuatu, residents were exposed to significant *Flavivirus* transmission. Compared to other seroprevalence studies, the marked difference between ZIKV immunity levels and previously published CHIKV seroprevalence rates in our study populations is surprising. We propose the timing of ZIKV and CHIKV emergence in relation to recurrent DENV outbreaks and the impact of seasonality as explanatory external factors for this observation. Our data add to the knowledge of arboviral epidemics in the SPR and contribute to a better understanding of virus spread, including external conditions with potential influence on outbreak dynamics. These data may support preventive and rapid response measures in the affected areas, travel-related risk assessment, and infection identification in locals and returning travelers.

## 1. Introduction

Arboviral infections are a common cause of disabling fever syndromes and a growing risk to global health [1,2]. They contribute to more than 17% of all infectious diseases worldwide and cause over 700,000 deaths per year [3]. In addition to their direct effect on human health, arboviruses also strongly compromise society and economy, temporarily stagnating it to some extent [4]. As they mainly circulate in tropical regions comprised of low-resource countries and affect already vulnerable populations less able to cope with the added burden, they have a disproportionate impact on the socio-economic sector of the world’s poorest societies [4]. During the past few decades, the dengue virus (DENV) became hyperendemic in many areas in the tropics and subtropics [5] and is now considered the most important arboviral disease in humans worldwide, causing 100–400 million infections with approx. 500,000 hospitalizations and 20,000–40,000 deaths annually [6,7,8,9]. 

DENV is a positive-strand RNA virus belonging to the *Flavivirus* genus [7]. It was first isolated in 1943 in Japan [10], but reports of possible outbreaks date back to 1779 [11]. DENV can be classified into four different serotypes (DENV-1 to DENV-4), and it is possible to become infected multiple times due to incomplete cross-protection [7]. While most infections are asymptomatic or manifest as a mild febrile illness, severe and potentially lethal hemorrhagic complications are mostly associated with subsequent infections (secondary infections) by other serotypes [7].

In addition to the immense disease burden caused by the circulating DENV alone, the health systems of many regions have been challenged in recent years by a previously lesser-known member of the *Flavivirus* genus—Zika virus (ZIKV). ZIKV was first isolated in 1947 from a rhesus monkey in the Zika forest in Uganda [12], and for six decades since its discovery, the virus remained confined to Africa and Asia, causing only sporadic outbreaks [13]. During large-scale outbreaks in the Pacific Region starting in 2007 [14] and the Americas starting in 2015 [15], ZIKV has shown its potential for a rapid population spread as well as its association with severe neurological complications in fetuses, neonates, and adults [16,17]. The teratogenic potential identified was an unprecedented feature in a mosquito-borne viral infection with dramatic implications for affected communities [18]. As a consequence, the World Health Organization declared ZIKV a public health emergency of international concern in February 2016 [19] and ZIKV is now considered the newest member of the TORCH pathogens (congenital infections that classically comprise toxoplasmosis, others (e.g., syphilis, hepatitis B), rubella, cytomegalovirus, and herpes simplex) [20]. Both, DENV and ZIKV are transmitted through bites of *Aedes* spp. mosquitoes, mainly *Aedes aegypti* and *Aedes albopictus*. 

Despite their significant effect on public health, society, economy, and social structures, exact information concerning the real local and global burden of arboviral diseases is often lacking [21,22]. One of the areas severely affected by DENV and ZIKV, but with limited epidemiological data, is the South Pacific Region (SPR) [2,23,24,25]. 

DENV is not endemic to the SPR, but since the 1970s, repeated introductions (mostly from Southeast Asia) caused numerous outbreaks of all four serotypes [26]. However, until recently, DENV circulation in the area was characterized by cyclical patterns, with a single serotype predominating for up to 5 years [27]. The first long-term co-circulation of several DENV serotypes was detected only in 2007, marking a substantial change in regional arboviral epidemiology [25]. In the same year, the first ZIKV outbreak outside of Africa and Asia was reported from the island Yap, part of the Federated States of Micronesia [14] and from 2013 onwards, ZIKV disseminated throughout the region, causing numerous outbreaks. 

**Figure 1:**
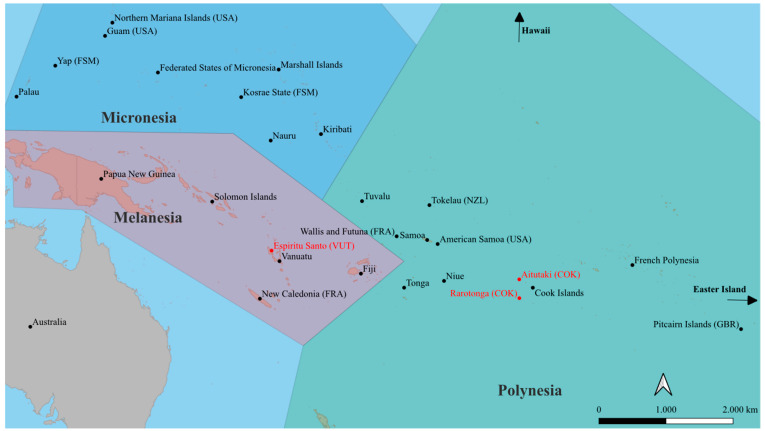
The South Pacific Region, overview with geographic subregions. Study settings in red. USA: United States of America, FSM: Federated States of Micronesia, FR: France, GBR: United Kingdom, NZL: New Zealand.

The SPR represents a special geographical environment: It is characterized by a vast area of open ocean with thousands of islands scattered in between, forming the 22 Pacific Island Countries and Territories (PICTs) [28] (Figure 1). These can be divided into the three sub-regions of Melanesia, Micronesia and Polynesia and are home to approximately 11.4 million Pacific islanders [22]. Many of the Pacific islands fall into the category of developing or least developed countries of the United Nations Development Programme and are among the countries most vulnerable to natural disasters [28]. The combination of tropical climate, archipelagic geography, the presence of potent vectors, immunologically naïve populations, the level of development, and frequent population flows, including millions of tourists per year, makes the region not only particularly vulnerable to arboviral epidemics, but also provides almost ideal outbreak conditions [22,23,25,29,30,31,32]. In addition to a high local burden of disease, it is therefore assumed that the SPR was one of the sources for the global spread of arboviruses observed in recent years, including DENV, ZIKV, and CHIKV [23,25], and more comprehensive knowledge of the arboviral situation in this region is of global interest.

With this study, we aim to further expand the knowledge on the epidemiology of three important pathogens. Our data can support local actions in affected areas by providing baselines for monitoring the evolution of seroconversion, which in turn may be used to evaluate prevention and outbreak control measures (e.g., vector control measures or the introduction of new vaccines). They can also contribute to an improved risk assessment in connection with travelling and the identification of imported infections and serve as a basis for further research. 

## 2. Materials and Methods

### 2.1. Ethics

This study was conducted in accordance with the Ethics Committee of the Medical Faculty of the Rheinisch-Westfaelische Technische Hochschule (RWTH) Aachen University (051/16_09/05/2016) and with the local authorities of the Cook Islands (Ref.: #16–16_31/05/2016) and Vanuatu (Ref.: MOH/DG 10/1/1-GKT/lr_27/06/2016).

### 2.2. Sample Collection and Analysis

Serum samples were collected in hospital laboratories of the island states of Vanuatu (outer island Espiritu Santo) during August 2016–January 2017 and the Cook Islands (main island Rarotonga and outer island Aitutaki) during January 2017–April 2017. Afebrile residents requiring venous blood testing within the hospitals’ normal diagnostic routine were asked to participate in the study, excluding tourists and short-term visitors. On each island, there was only one hospital laboratory. After obtaining written informed consent, basic epidemiological information was collected via questionnaires and interviews. 

In total, 626 serum specimens (350 from Espiritu Santo = 1% of the resident population, 208 from Rarotonga = 2% of the resident population and 68 from Aitutaki = 4% of the resident population) were collected and analyzed for anti-DENV-specific antibodies (IgG) using a recombinant-antigen-based indirect enzyme-linked immunosorbent assay (ELISA) (EUROIMMUN Lübeck, Germany (EI 266b-9601 G); sensitivity: 99%; specificity: 96%). Among those specimens, 465 randomly chosen samples (197 from Espiritu Santo, 208 from Rarotonga and 60 from Aitutaki) were further analyzed for anti-ZIVK specific antibodies (IgG) (ELISA, EUROIMMUN Lübeck, Germany (EI 2668-9601 G); sensitivity: 76%; specificity: 100%). Signal-to-cutoff ratios were calculated following the manufacturer’s instructions. Test results were defined as “positive”, “negative” or “equivocal” if in between the threshold values. The serological tests were conducted according to the manufacturer’s recommendations with quality control measures (positive/negative controls and calibration samples) included with each plate to assess the validity of the results.

Ten randomly chosen samples from all three islands were further assessed by indirect immunofluorescent assays (IIFA). IIFA for DENV and ZIKV was performed using the EUROIMMUN Arbovirus Fever Mosaic 2 EUROPattern testing for IgG. Tests were carried out according to the manufacturer’s instructions with an initial dilution of 1:20. In order to minimize false-positive results, a further dilution was performed if the test result was positive. DENV titers were considered as solely positive if they were at least 4-fold higher than ZIKV titers and vice versa.

### 2.3. Statistics

Associations between seroprevalence levels and test collective as well as between ELISA test results and gender were performed using the Chi^2^-Test. For all comparisons the significance level was set to 5%; due to the explorative nature of the investigation, no adjustment to the significance level was made. Results were reported as a percentage and two-sided *p*-values. 

As comparing rates between different geographical areas is usually more representative when taking into account differences in the gender and age structure of the individual populations, we mathematically adjusted the different populations by a direct standardization to achieve the same gender and age structure as a standard population. This step was carried out in order to compare the results of the test populations with each other, with the entire test population serving as the standard population. To transfer the results to a higher-level population and to enable estimations and comparisons of community immunity-levels, raw data were directly standardized by age (10-year age groups; excluding the age group of 0–9 years due to low numbers) and gender according to the standard populations “total resident population” (Cook Islands) and “total population living in private households” (Vanuatu). The respective reference data were published in the Cook Islands Census of Population and Dwellings, 2011 [33] and in the Vanuatu Post-Tropical Cyclone Pam Mini-Census, 2016 [34], respectively (further referred to as “resident population” or “residents”). Estimates of the immunity in the different collectives were accompanied by a 95% confidence interval (CI).

### 2.4. Data Collection, Data Processing, and Visualization

Information concerning outbreaks was obtained from publications accessed via PubMed or from local surveillance data. Data regarding the history of the Cook Islands ZIKV outbreak consists of probable and confirmed cases and derives from the archives of the Pacific Public Health Surveillance Network. All analyses were performed with Microsoft Excel Office 365 and IBM SPSS Statistics 21. Graphs were created using Microsoft PowerPoint Office 365 and QGIS Geographic Information System version 3.32. The base layer of the maps was made with Natural Earth, free vector and raster map data. 

## 3. Results

**Figure 2:**
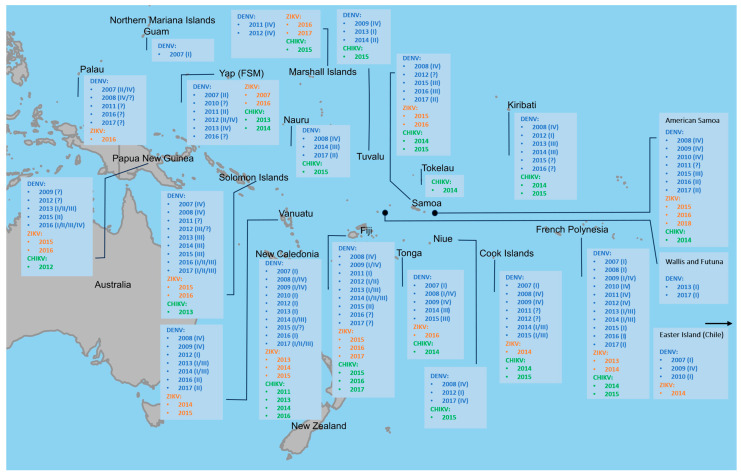
Circulation of DENV, ZIKV and CHIKV in the SPR 2007–2017, locations outside the map area indicated by arrows.

Figure 2 shows a summary of DENV, ZIKV and CHIKV circulation in the SPR during 2007–2017 (see Table A1, Table A2 and Table A3). 

**Figure 3:**
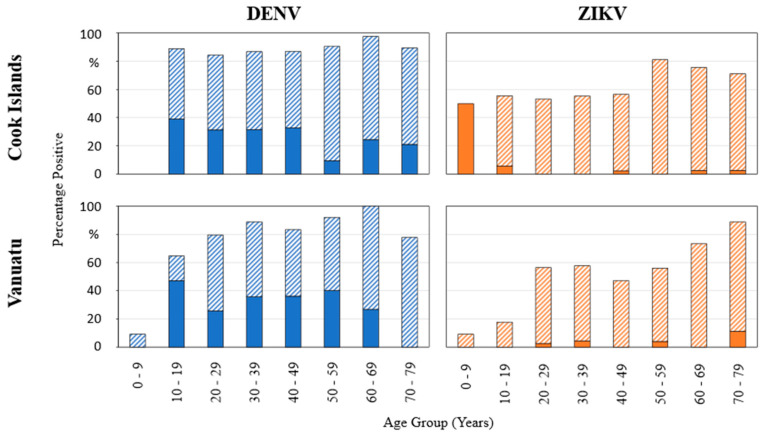
Distribution of seroprevalence for Flaviviruses across 10 year age groups. Blue: DENV, orange: ZIKV. Single-color-filled area represents samples that were positive for the respective virus alone; hatched shading indicates samples that were positive for both Flaviviruses (DENV and ZIKV). Refer to [35] for a comparable presentation of a seroprevalence study in the Solomon Islands.

Study populations were defined according to their islands of origin—the Cook Islands and Vanuatu—with the Cook Islands collective subdivided into two further subgroups (Rarotonga and Aitutaki). All specimens were tested for anti-DENV antibodies, and the majority was subsequently further analyzed for anti-ZIKV and anti-CHIKV antibodies (Table A4). Detailed test results can be found in the supplementary file (S1). Of all specimens tested for antibodies against both arboviruses (268 from the Cook Islands, 197 from Vanuatu), there were significantly more Cook Island samples than Vanuatu samples that tested positive for either *Flavivirus* (91% and 83%, respectively; Chi^2^-Test *p*-value: 0.011). Within those samples positive for *Flaviviruses*, one quarter (Cook Islands) and approx. one third (Vanuatu) had antibodies solely for DENV, while in both test collectives, five samples were tested positive for anti-ZIKV but negative for DENV. In general, seroprevalence of *Flaviviruses* was largely stable across all age groups (Figure 3, Table A5). However, the prevalence of individuals positive for both *Flaviviruses* especially increased by age, peaking in the 50–59 years group for the Cook Islands and in the 70+ years group in Vanuatu. 

**Figure 4:**
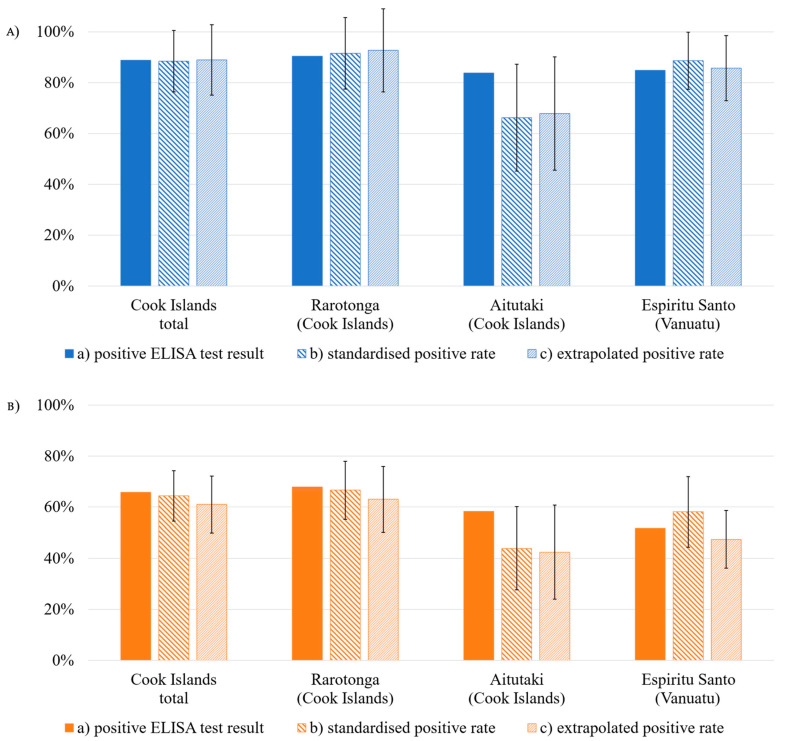
Rates of positive ELISA test results, seropositivity rates standardized by gender and age and seropositivity rates extrapolated to the total resident population (for further information see Table A6). Black whiskers show 95% CI relative to standardized and extrapolated rate. (**A**) DENV, (**B**) ZIKV.

Analyzed individually (Figure 4, Table A6), 89% of all Cook Islands test sera and 85% of the Vanuatu specimen tested positive for former DENV contact. Further subdividing the Cook Islands test group, 90% of the Rarotonga collective was identified as being positive, compared to 84% of the Aitutaki serum samples. Anti-ZIKV antibodies were found in 66% of the Cook Island test collective and in 52% of the Vanuatu specimens. 

**Figure 5:**
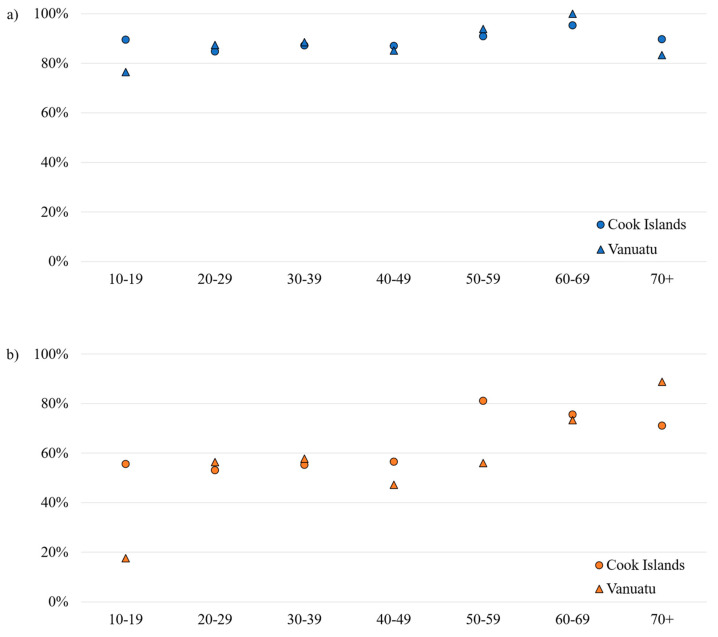
Positive test results by 10 year age groups. (**a**) DENV, (**b**) ZIKV.

Using the Chi^2^-Test, it could be shown that there was no significant association between ELISA test results and gender either for anti-DENV antibodies (*p*-value: 0.349 (Cook Islands), *p*-value: 0.837 (Vanuatu)) or for anti-ZIKV (*p*-value: 0.660 (Cook Islands), *p*-value: 0.302 (Vanuatu)). Regarding seropositivity rates across 10 year age groups (age group 0–9 years was excluded due to low case numbers), in both test collectives, DENV seroprevalence remained stable on a high level, while ZIKV seropositivity peaked in the age groups 50–70+ years (Figure 5).

To allow for a direct comparison of our test collectives’ seroprevalence levels and to test for significant differences between them, data were standardized by age and gender (Figure 4, Table A6). Standardized DENV immunity rates account for 89% in both test collectives without significant difference (Chi^2^-Test *p*-value: 0.945). The difference was also shown to be of no statistical significance in standardized ZIKV seropositivity rates (Cook Islands: 64%; Vanuatu: 58%) (Chi^2^-Test *p*-value: 0.179). Analyzing the two Cook Islands subpopulations, standardized seropositivity rates for both arboviruses were significantly higher in the Rarotonga test collective (DENV: 92%, ZIKV: 67%) than in the Aitutaki test group (DENV: 66%, ZIKV: 44%) (Chi^2^-Test *p*-value: <0.001 (DENV), *p*-value: 0.001 (ZIKV)). 

Extrapolated to the total population (>9 years), calculated immunity levels in the Cook Islands sum up to 89% for DENV and 61% for ZIKV (Figure 4, Table A6). In the local population of Vanuatu (>9 years), seroprevalence rates of 86% for DENV and 47% for ZIKV were calculated (Figure 4, Table A6). For both viruses, differences between the two PICTs were shown to be of statistical significance (*p*-values: <0.001 (DENV), *p*-value: <0.001 (ZIKV)). Regarding the two islands Rarotonga and Aitutaki, extrapolated seropositivity of the local population (>9 years) amounts to 93% and 68% for DENV and to 63% and 42% for ZIKV, respectively, with a statistically significant difference (*p*-values: <0.001 (DENV), *p*-value: <0.001 (ZIKV)). 

**Figure 6:**
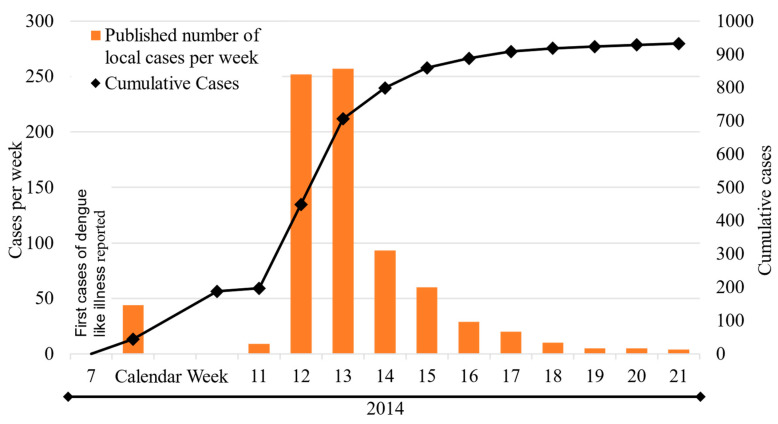
History of the 2014 Cook Islands ZIKV outbreak as published by local syndromic surveillance systems.

During the Cook Islands ZIKV epidemic, 932 clinically diagnosed cases were reported, of which 49 were confirmed via laboratory testing [32,36]. The history of the Cook Islands ZIKV epidemic as reported by local surveillance systems is displayed in Figure 6. The vast majority of all cases occurred on the main island of Rarotonga, and there were no severe complications or associated hospitalizations [36]. Detailed information concerning the index case is missing, but there is evidence that ZIKV was introduced to the Cook Islands from a returning traveler from French Polynesia where the virus was circulating at that time [37]. This hypothesis is supported by genetic analysis showing the Cook Island ZIKV strain to be closely related to French Polynesia isolates [38]. Considering our extrapolated results, numbers suggest that only a small proportion of all infections was reported and implies a calculated case detection rate of 9%. Equally detailed data for the Vanuatu ZIKV outbreak are not available.

We further employed indirect immunofluorescence assays for further testing of ten randomly chosen specimens from all three islands. Of all ten specimens tested with IIFA, three showed discordant results concerning anti-DENV positivity and two concerning anti-ZIKV positivity (Table 1). Interestingly, in all cases, IIFA was positive, while ELISA showed negative test results; there was no case of false-positive ELISA test results identified. Of all ten patients tested positive for anti-DENV antibodies, two were confirmed negative for anti-ZIKV antibodies.

## 4. Discussion

We found that in both settings, the Cook Islands and Vanuatu, residents have been exposed to substantial arboviral transmission. Results on the seroprevalence of CHIKV in the same study populations have been published and discussed previously [39]. We will therefore first focus on the detected *Flavivirus* seroprevalence rates and subsequently examine the differences observed in relation to CHIKV immunity levels. 

**Figure 7:**
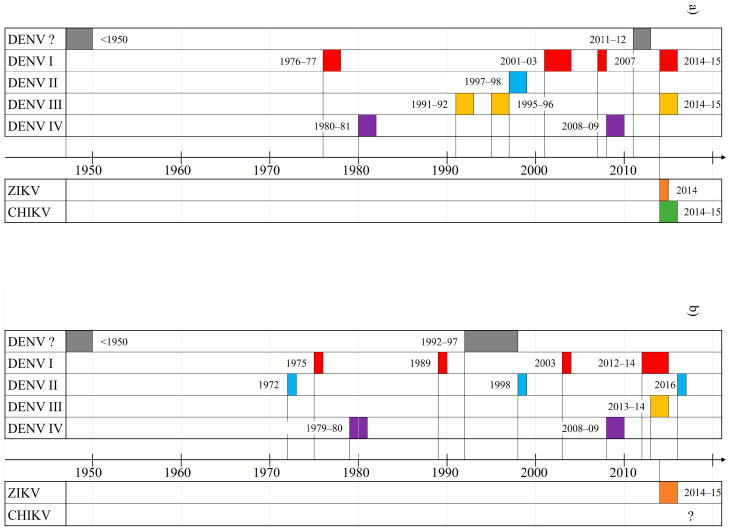
History of DENV, ZIKV and CHIKV outbreaks (**a**) in the Cook Islands and (**b**) in Vanuatu. Grey: DENV serotype unknown, red: DENV-1, blue: DENV-2, yellow: DENV-3, purple: DENV-4, orange: ZIKV, green: CHIKV.

Reactivity to at least one *Flavivirus* was observed in 91% (Cook Islands) and 83% (Vanuatu) of all tested sera with 64% and 49%, respectively, even showing evidence of past exposure to both DENV and ZIKV. Especially in the Vanuatu test group, *Flavivirus* seropositivity rates increased rapidly with age before reaching a steady high level. This is indicative of a continuously intense and long-lasting exposure of the population, with older people being more likely to have been exposed throughout their lifetime. Findings are consistent with epidemiological data demonstrating high-frequency outbreaks of DENV in both settings (Figure 7, Table A7). However, statistically significant differences between the two Cook Islands subgroups (Rarotonga and Aitutaki) depict regional heterogeneity, probably as a result of the scattered archipelagic geography. As the same effect has been shown for CHIKV [39], it can be assumed that in the SPR, arboviral diseases might have a greater impact on the populations of the main islands compared to outer islands. The current policy of reducing domestic flight connections between individual islands during an epidemic could further intensify this effect.

Examined individually, as much as 89% and 66% of the Cook Islands sera as well as 85% and 52% of the Vanuatu specimen were tested positive for anti-DENV and anti-ZIKV antibodies. Surprisingly, in both test collectives, ZIVK seroprevalence rates peak in the older age groups despite the absence of recurrent epidemics. It could be assumed that this could be due to *Flavivirus* cross-reactivity as ELISA tests are known to have high sensitivity but to be prone to cross-reactivity with other arboviruses lowering specificity [40,41]. The bias caused by potential false-positive test results can be overcome through control with further test methods such as, for example, IIFA. These tests are time-consuming, labor-intensive, and expensive and are therefore not as amenable to testing large numbers of sera as the ELISA is. For these reasons, in our study, only a small number of specimens could be analyzed. Surprisingly, we did not identify any false-positive test results in the ELISA analysis, but conversely found three (DENV) and two (ZIKV) specimens showing evidence for former virus contact in the IIFA which had tested negative in the preceding ELISA analysis. The number of specimens tested with IIFA was small and findings are presumably not representative for the whole test collective. The results were, however, unexpected and tend to contradict the high false-positive rate of the ELISA. On the other hand, false-negative ELISA test results could possibly represent a time-dependent decline in antibody-levels already observed in other surveys [42]. Though results must be interpreted with care, it is therefore possible that seroprevalence levels directly after the respective outbreaks were even higher than those detected in our survey. The effects of reduced antibody titers on immunity and severity of disease in case of reinfection remain unclear [42,43]. We could not identify significant differences in seroprevalence rates between male and female probands either in the Cook Islands or in the Vanuatu test collective, suggesting that there is no major gender-related behavioral difference reducing or increasing the risk of exposure. 

**Figure 8:**
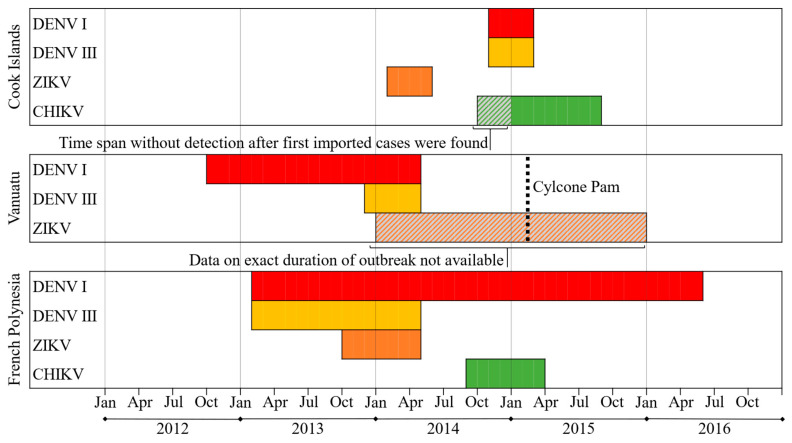
Timeline of DENV, ZIKV and CHIKV outbreaks in the Cook Islands, Vanuatu, and French Polynesia 2012–2016, including cyclone “Pam”. Bars representing the duration of the outbreak; red: DENV-1, yellow: DENV-2, orange: ZIKV, green: CHIKV, dashed color filling: data on exact duration not available.

Evidence for former CHIKV infection has shown to be much lower in both settings, with 30% (Cook Islands) and 8% (Vanuatu) of seropositivity [39]. While standardized seroprevalence levels for DENV and ZIKV did not show significant differences between the two main test collectives, the CHIKV immunity rates in the Vanuatu population were significantly lower. Therefore, seroprevalence levels against CHIKV show both (i) major deviations between the two study settings and (ii) significantly lower rates compared to the other arboviruses examined within the same study populations. Epidemiological studies require the investigation of potential driving factors of arboviral spread. A detailed comparison of relevant environmental and social data in the study areas can be found in [39]. While possible explanations for the differences in CHIKV immunity levels between the Cook Islands and Vanuatu (i) including the presence or absence of secondary vectors like *Ae. albopictus* and *Ae. polynesiensis*, human population densities, mobility of the resident population, and tourism have already been discussed [39], in this paper, we focus on the difference in seroprevalence between ZIKV and CHIKV within the same test groups (ii). This discrepancy is surprising because, unlike DENV, the epidemics of ZIKV and CHIKV were virgin soil outbreaks transmitted by the same vectors and occurred in the same locations in 2014/2015. In fact, our results differ from some other island surveys conducted in the SPR and the Caribbean which found CHIKV seroprevalences to be higher compared to ZIKV immunity levels [44,45,46]. Common explanations for this observation include a higher viral load in patients with CHIKV that could result in increased transmission rates, and possibly cross-protective pre-existing DENV-antibodies which may have limited ZIVK spread [46]. In terms of viral factors, however, studies have shown that ZIKV and CHIKV have similar reproduction numbers [47,48,49] and do not vary significantly in transmissibility when assessed under the same conditions [50]. As this suggests that the epidemic dynamics are determined less by differences between the viruses than by external factors, we focus on differences in external conditions during both outbreaks. One of these is the timing of the ZIKV and CHIKV epidemics in the context of recurrent DENV outbreaks in the SPR: During the emergence of the two new entities, the SPR experienced outbreaks of DENV-1&-3 in various locations (Figure 2, Table A1, Table A2 and Table A3). In the Cook Islands, however, ZIKV occurred prior to the 2014/2015 DENV-1&-3 epidemic, while CHIKV emerged during DENV circulation (Figure 8, Table A8). In the neighboring French Polynesia, where CHIKV seroprevalence rates (76%) were shown to outnumber those of ZIKV (49%) [44,45], the 2013/2014 Zika fever epidemic happened during the DENV-1&-3 co-epidemic, while CHIKV emerged only after transmission of DENV-3 had already ended (Figure 8, Table A8). Vanuatu, on the other hand, reported an occurrence of DENV serotype 1 and/or 3 between October 2012 and April 2014 (Figure 8, Table A8). At this time, CHIKV circulated in its neighboring countries New Caledonia (2013, [51]) and the Solomon Islands (2013, [52]) (Table A3), but despite an estimated high risk of virus importation [51], CHIKV transmission was not detected in Vanuatu. In general, there is evidence that viral co-infection has very little effect on the vector competence of *Ae. aegypti* [53]. However, in the case of CHIKV, co-infection with DENV (Serotype 2) has shown to significantly reduce transmission rates by 27% compared to mosquitoes that were only exposed to CHIKV [53]. Yet the reasons for reduced transmission during viral co-circulation are not necessarily limited to competition for common vectors. Other possible influencing factors include a change in the behavioral pattern of the affected population (enhanced vigilance and increased individual mosquito protection during an epidemic/sick people spending more time indoors or under mosquito nets), and the effect of introduced vector control measures. We therefore suggest that in our study locations, circulating DENV might have limited CHIKV transmission due to competition for vectors and hosts, leading to reduced CHIKV seroprevalence rates. ZIVK, on the other hand, occurred prior to (Cook Islands) and after (Vanuatu) the DENV epidemics and could therefore spread free from other competitive arboviruses and infect large parts of the population. In addition to these considerations, it is possible that the transmission of ZIKV in Vanuatu was exacerbated by another external factor: tropical cyclone “Pam”, which hit the island state in March 2015 (Figure 8). The category five superstorm left thousands of people homeless and caused widespread destruction in the island state [34] with tons of debris that likely served as a breeding ground for mosquitoes. In the aftermath of cyclone “Pam”, medical teams reported several cases of “pink eye” to the WHO [54], and the first ZIKV infection was reported in Vanuatu just weeks after the natural disaster [37,55]. 

Another possible explanation for the observed discrepancies in seroprevalence in our study populations is seasonality. As detailed data from outbreaks in Vanuatu are lacking, the available information allows for a direct comparison between the ZIKV and CHIKV epidemics only for the Cook Islands: both viruses were introduced to the Cook Islands in the same year (2014), but at different times of the year (February (ZIKV) versus October/November (CHIKV)). While the ZIKV epidemic had a sudden, intense but short course (Figure 6), the CHIKV outbreak developed more slowly and shallowly, with a latency period of several weeks between the first imported cases and the onset of high-level local transmission [39]. Data on possible low-level circulation during this latency period are not available, but even based on the first known cases, the CHIKV epidemic was twice as long in terms of duration. However, a correlation between seasonality and the observed different outbreak dynamics and variations in seroprevalence can only be suspected and would require additional verification by more complex modeling including vector and host factors (e.g., extrinsic incubation period at different temperatures or seasonal travel behavior). 

As with many seroprevalence surveys, limitations result from the study design [21]: representativeness is lowered using serum samples collected from hospital patients (convenience sampling), rather than from the general population. Compared to the census data, both study populations show some deviations from the general population: Our test populations have a higher median age (Cook Islands: 49 years/Vanuatu 32 years) than the census populations (Cook Islands: 29 years/Vanuatu 20 years). The age group 0–9 years is especially underrepresented, and we had to exclude it from standardization and extrapolation. Furthermore, due to antenatal care, women are overrepresented. We want to emphasize that we could only include residents of a few selected islands in our study. Even though the included islands are among the most populous of the two island states, they are composed of significantly more (inhabited) islands whose residents are not mapped in this study. Regarding the isolated nature of our study settings, this could lead to false estimations concerning the seroprevalence levels on islands not depicted in this survey and extrapolated seropositivity rates should be interpreted with caution. In addition, there is little information available on the ZIKV outbreak in Vanuatu.

Although seroprevalence rates detected in our survey are consistent with epidemiologic data, another limiting factor which cannot be ruled out is the risk of false-positive and false-negative test results as our interpretation is ELISA-based and seroneutralization tests have not been performed. Among the few IIFAs, we did not detect any false-positive ELISA test results. The number of samples tested with IIFA is small though and results are not representative of the entire test collective. Further, result interpretation of IIFA is subjective [41]. However, data indicating a low probability of cross-reactions between dengue and Zika in ELISA tests derive from French Polynesia: in a serological survey conducted with sera collected prior to the Zika epidemic, only 0.8% of the individuals were seropositive for ZIKV, despite high immunity levels against DENV [56]. As the IIFAs have shown, there is also the possibility of falsification by false-negative ELISA test results, which we hypothesize might be due to a decline in seroprevalence rates over time. Waning immunity has been reported by several authors [57,58,59], but detailed information on the underlying mechanisms and consequences for subsequent epidemics is currently not available. Further, we did not perform tests to differentiate the detected seroprevalence rates regarding the individual DENV serotypes. It is therefore not possible to draw a conclusion about the degree of exposure or protection of our study populations against the individual dengue viruses. Thus, our test results must be interpreted with care and should not mislead clinical diagnosis.

To summarize, our seroprevalence data complement the current epidemiological knowledge and show that in both island states, the Cook Islands and Vanuatu, residents have been exposed to substantial arboviral transmission though with significant regional heterogeneity. Focusing on *Flaviviruses*, results illustrate the immense disease burden caused by DENV in the SPR and highlight the dimensions of the individual ZIKV epidemics in 2014 and 2015. Our results are generally in good agreement with surveillance reports; however, they also support the hypothesis that surveillance systems are particularly useful for detecting outbreaks and providing a general overview of the epidemic situation, but do not reflect the actual disease burden in a population [21]. This is as they often remain sporadic, incomplete, or delayed and tend to underestimate the extent of an epidemic [22]. With its calculated case detection rate of 9%, the proportion of cases diagnosed during the ZIKV epidemic in the Cook Islands is below the rate of diagnosed CHIKV cases (18%) in the same population [39]. Differences in reporting rates for the two diseases have been observed before [50] and are consistent with a much higher symptomatic rate of CHIKV infections (more than 80%) compared to ZIKV infections (around 20%) [60,61]. Detected seroprevalence rates are contrary to this and show significantly higher rates for ZIKV than for CHIKV in both our study settings. 

The current gap in knowledge about the real dimensions of arboviral outbreaks is critical for diagnosis, vector control, vaccine introduction, the identification of target populations, avoidance of sub-optimal cost-effectiveness, and (in the case of DENV) a potential increased risk for severe cases if the vaccine is not targeted appropriately [62,63]. Therefore, seroprevalence studies such as this one provide important baseline data that can be used to monitor the evolution of seroconversion, decision making, and to assess the risk of future epidemics in the event of virus reintroduction. However, to provide improved forecasts of virus transmission as well as a retrospective evaluation of the effectiveness of preventive and outbreak control measures, such seroepidemiological approaches need to be employed in many other settings. Equally important are detailed investigations aiming at disentangling the complex interplay of ecological, environmental, and social factors on the one hand and dynamics in arboviral transmission on the other hand. In addition, further research on vaccination, vectors and vector control methods, the effects of decreasing seroprevalence rates over time in populations that have already experienced virus transmission, efficient case detection, and public health campaigns is needed to address the growing threat of arboviruses to global health.

## Figures and Tables

**Table 1:** Results of indirect immunofluorescence assay, results discordant to ELISA test results in red font.

Specimen	DENV		ZIKV	
	ELISA	IIFA	ELISA	IIFA
**A**	positive	positive	positive	positive
**B**	positive	positive	negative	positive
**C**	positive	positive	positive	positive
**D**	positive	positive	negative	negative
**E**	positive	positive	positive	positive
**F**	positive	positive	positive	positive
**G**	negative	positive	negative	negative
**H**	positive	positive	negative	positive
**I**	negative	positive	positive	positive
**J**	negative	positive	positive	positive

## Data Availability

The original contributions presented in the study are included in the article/Appendix A material, further inquiries can be directed to the corresponding author/s. Data concerning CHIKV were published in full in [39] including the Supplementary Material.

## Supplementary Materials

The following supporting information can be downloaded at: https://www.mdpi.com/article/10.3390/v16050807/s1.

## Appendix A

**Table A1:** DENV outbreaks in the SPR 2007–2017.

Year	Location	Serotype/Genotype	References
2007	Tonga	DENV-1	[64]
2007	Cook Islands	DENV-1	[25]
2007	French Polynesia	DENV-1	[25,65]
2007	Guam	DENV-1	[25]
2007	New Caledonia	DENV-1	[25]
2007	Easter Island (Chile)	DENV-1	[25]
2007	Federated States of Micronesia	DENV-2	[25]
2007	Palau	DENV-2/4	[25]
2007	Solomon Islands	DENV-4	[25,64]
2008	French Polynesia	DENV-1	[25,65]
2008	Tonga	DENV-1/4	[25,64]
2008	New Caledonia	DENV-1/4	[25,64]
2008	Nauru	DENV-4	[64]
2008	Kiribati	DENV-4	[25,64]
2008	Samoa	DENV-4	[25,64]
2008	American Samoa	DENV-4	[25,64]
2008	Palau	DENV-4/?	[25,64]
2008	Cook Islands	DENV-4	[25,64]
2008	Fiji	DENV-4	[25,64]
2008	Niue	DENV-4	[64]
2008	Solomon Islands	DENV-4	[64]
2008	Vanuatu	DENV-4	[64]
2009	New Caledonia	DENV-1/4	[25,64]
2009	French Polynesia	DENV-1/4	[25,64,65]
2009	Fiji	DENV-1/4	[25]
2009	Vanuatu	DENV-4	[25,64]
2009	Cook Islands	DENV-4	[25]
2009	Tuvalu	DENV-4	[25]
2009	Tonga	DENV-4	[25]
2009	American Samoa	DENV-4	[25]
2009	Easter Island (Chile)	DENV-4	[64]
2009	Papua New Guinea	DENV-?	[25]
2010	New Caledonia	DENV-1	[25]
2010	Easter Island (Chile)	DENV-1	[25]
2010	American Samoa	DENV-4	[25]
2010	French Polynesia	DENV-4	[25,65]
2010	Federated States of Micronesia	DENV-?	[25]
2011	Federated States of Micronesia	DENV-2	[25]
2011	Marshall Islands	DENV-4	[25]
2011	Fiji	DENV-1	[25]
2011	Palau	DENV-?	[25]
2011	Solomon Islands	DENV-?	[25]
2011	French Polynesia	DENV-4	[65]
2011	Cook Islands	DENV-?	[25]
2011	American Samoa	DENV-?	[25]
2012	Niue	DENV-1	[25,32]
2012	Kiribati	DENV-1	[25,32]
2012	Fiji	DENV-1/2	[25,32]
2012	New Caledonia	DENV-1	[25,32]
2012	Federated States of Micronesia	DENV-2/4	[25,32]
2012	Solomon Islands	DENV-3/?	[25,32]
2012	Marshall Islands	DENV-4	[25]
2012	Papua New Guinea	DENV-?	[25]
2012	Samoa	DENV-?	[25]
2012	Vanuatu	DENV-1	[32]
2012	French Polynesia	DENV-4	[65]
2012	Cook Islands	DENV-?	[25]
2013	Wallis and Futuna	DENV-1	[32]
2013	Tuvalu	DENV-1	[25]
2013	New Caledonia	DENV-1	[25,32]
2013	French Polynesia	DENV-1/3	[25,27,32,65]
2013	Papua New Guinea	DENV-1/2/3	[25]
2013	Fiji	DENV-1/3	[25,32]
2013	Vanuatu	DENV-1/3	[25,32]
2013	Solomon Islands	DENV-3	[25,27,32,66]
2013	Kiribati	DENV-3	[25,32]
2013	Federated States of Micronesia	DENV-4	[25]
2014	Fiji	DENV-1/2/3	[25,32]
2014	New Caledonia	DENV-1/3	[25,32]
2014	French Polynesia	DENV-1/3	[22,25,32,65]
2014	Tuvalu	DENV-2	[25,32]
2014	Tonga	DENV-3	[22,25,32]
2014	Nauru	DENV-3	[25,32]
2014	Kiribati	DENV-3	[25]
2014	Solomon Islands	DENV-3	[25,32]
2014	Vanuatu	DENV-1/3	[25,32]
2014	Cook Islands	DENV-1/3	[22]
2015	Fiji	DENV-2	[22]
2015	Cook Islands	DENV–1/3	[22]
2015	New Caledonia	DENV-1/?	[22]
2015	Tonga	DENV-3	[22]
2015	Kiribati	DENV-?	[22]
2015	Solomon Islands	DENV-3	[22]
2015	French Polynesia	DENV-1	[22,65]
2015	American Samoa	DENV-3	[22]
2015	Samoa	DENV-3	[22]
2015	Papua New Guinea	DENV-2	[22]
2016	Papua New Guinea	DENV-1/2/3/4	[22]
2016	Kiribati	DENV-?	[22]
2016	Fiji	DENV-?	[22]
2016	French Polynesia	DENV-1	[65]
2016	Federated States of Micronesia	DENV-?	[22]
2016	New Caledonia	DENV-1	[22]
2016	Vanuatu	DENV-2	[22]
2016	Palau	DENV-?	[22]
2016	Samoa	DENV-3	[22]
2016	American Samoa	DENV-2	[22]
2016	Solomon Islands	DENV-1/2/3	[22]
2017	New Caledonia	DENV-1/2/3	[22]
2017	Vanuatu	DENV-2	[22]
2017	Palau	DENV-?	[22]
2017	American Samoa	DENV-2	[22]
2017	Solomon Islands	DENV-1/2/3	[22]
2017	French Polynesia	DENV-1	[65]
2017	Fiji	DENV-?	[22]
2017	Nauru	DENV-2	[22]
2017	Niue	DENV-4	[22]
2017	Samoa	DENV-2	[22]
2017	Wallis and Futuna	DENV-1	[22]

**Table A2:** Spread of ZIKV in the SPR.

Year	Location	Genotype	References
April 2007–July 2007	Federated States of Micronesia (Yap)	Asian	[14,16,25,67]
October 2013–April 2014	French Polynesia	Asian	[16,25,68,69]
November 2013	New Caledonia	Asian	[25,38,70]
January 2014–May 2014	Easter Island, (Chile)	Asian	[25,71]
January 2014–August 2014	New Caledonia	Asian	[16,25,37,38,70]
February 2014–May 2014	Cook Islands	Asian	[25,32,37,38]
2014 (prior to July)–exact time and duration unknown	Vanuatu (exported to New Zealand and New Caledonia)	unknown	[36,37]
January 2015–August 2015	New Caledonia	Asian	[37,72]
February 2015–May 2015 (evidence for sustained transmission until December 2016)	Solomon Islands	Asian	[35,37,72,73]
March/April 2015–exact time and duration unknown	Vanuatu	Asian	[22,37,38,72]
May 2015December 2015February 2016	Papua New Guinea		[22,72]
July 2015–October 2016(evidence for sustained transmission until June 2017)	Fiji	Asian	[22,38,72,74]
September 2015–May 2016	Samoa	Asian	[22,72,75]
December 2015–ongoing in November 2016	American Samoa	Asian	[16,72]
January 2016–June 2016	Tonga	Asian	[22,72]
February 2016–April 2016	Marshall Islands		[22,72]
February 2016–ongoing in November 2016	Federated States of Micronesia (Kosrae)		[72]
November 2016	Palau		[22,72]
January 2017	Marshall Islands		[22]
June 2018–November 2018	American Samoa		[22]

**Table A3:** Spread of CHIKV in the SPR.

Year	Location	Genotype	References
Februar 2011–June 2011	New Caledonia	Asian	[25,76,77]
June 2012–November 2012	Papua New Guinea	ECSA (IOL) (E1-A226V)	[25,32,78]
April 2013–exact duration unknown	Solomon Islands	Asian	[52]
February 2013–November 2013	New Caledonia	Asian	[51]
August 2013–August 2014	Federated States of Micronesia (Yap)	Asian	[25,32,79]
February 2014–ongoing in September 2014	Tonga	Asian	[25,32,74,80]
July 2014–March 2015	Samoa	Asian	[22,25,32,81,82]
July 2014–October 2014	Tokelau		[22,25,32,80]
June 2014–December 2014	American Samoa	Asian	[22,25,32,80,82,83]
September 2014–March 2015	French Polynesia	Asian	[50,80]
October 2014 (first imported cases)–August 2015	Cook Islands		[22,84]
October 2014–December 2014	New Caledonia		[22,25,85]
February 2015–October 2015	Marshall Islands		[22,86]
December 2014–March 2015	Kiribati	Asian	[22,82,87]
February 2015–exact duration unknown	Niue		[88]
March 2015–June 2016 (evidence for sustained transmission until June 2017)	Fiji	Asian	[74]
June 2015	Nauru		[22,89]
October 2015–November 2015	Tuvalu		[22,90]
January 2016	New Caledonia		[22]

**Table A4:** Specimen tested for anti-DENV, anti-ZIKV, and anti-CHIKV antibodies.

	Total Number	Specimen from the Cook Islands (Total)	Specimen from Rarotonga	Specimen from Aitutaki	Specimen from Vanuatu
**Tested for anti-DENV antibodies**	626	276	208	68	350
**Tested for anti-DENV antibodies and subsequently for anti-ZIKV and anti-CHIKV antibodies**	465	268	208	60	197

**Table A5:** Distribution of Flavivirus seroprevalence across 10 year age groups.

	Total Number Tested		Anti-Flavivirus ELISA Positive		Only Anti-DENV ELISA Positive		Only Anti-ZIKV ELISA Positive		Anti-DENV and Anti-ZIKV Positive	
10-Year Age Group	Cook Islands	Vanuatu	Cook Islands	Vanuatu	Cook Islands	Vanuatu	Cook Islands	Vanuatu	Cook Islands	Vanuatu
**0–9**	2	11	1 (50.0%)	1 (9.1%)	0 (0.0%)	0 (0.0%)	1 (50.0%)	0 (0.0%)	0 (0.0%)	1 (9.1%)
**10–19**	18	17	17 (94.4%)	11 (64.7%)	7 (38.9%)	8 (47.1%)	1 (5.6%)	0 (0.0%)	9 (50.0%)	3 (17.7%)
**20–29**	32	39	27 (84.4%)	32 (82.1%)	10 (31.3%)	10 (25.6%)	0 (0.0%)	1 (2.6%)	17 (53.1%)	21 (53.9%)
**30–39**	38	45	33 (86.8%)	42 (93.3%)	12 (31.6%)	16 (35.6%)	0 (0.0%)	2 (4.4%)	21 (55.3%)	24 (53.3%)
**40–49**	46	36	41 (89.1%)	30 (83.3%)	15 (32.6%)	13 (36.1%)	1 (2.2%)	0 (0.0%)	25 (54.4%)	17 (47.2%)
**50–59**	53	25	48 (90.6%)	24 (96.0%)	5 (9.4%)	10 (40.0%)	0 (0.0%)	1 (4.0%)	43 (81.1%)	13 (52.0%)
**60–69**	41	15	41 (100.0%)	15 (100.0%)	10 (24.4%)	4 (26.7%)	1 (2.4%)	0 (0.0%)	30 (73.2%)	11 (73.3%)
**70 and >70**	38	9	35 (92.1%)	8 (88.9%)	8 (21.1%)	0 (0.0%)	1 (2.6%)	1 (11.1%)	26 (68.4%)	7 (77.8%)
**Total**	268	197	243 (90.7%)	163 (82.7%)	67 (25.0%)	61 (31.0%)	5 (1.9%)	5 (2.5%)	171 (63.8%)	97 (49.2%)

**Table A6:** Rates of positive ELISA test results, seropositivity rates standardized by gender and age and seropositivity rates extrapolated to the total resident population with 95% CI. (a) DENV, (b) ZIKV.

(a)				
	**DENV**			
	**Cook Islands (Total)**	**Rarotonga**	**Aitutaki**	**Vanuatu**
**Number of positive ELISA Test results (% of test population)**	245 (88.8%)	188 (90.4%)	57 (83.8%)	297 (84.9%)
**Standardized immunity rates (95% CI)**	88.5% (76.4–100.5)	91.6% (77.5–105.6)	66.3% (45.2–87.3)	88.6% (77.4–99.9)
**Extrapolates immunity rates (95% CI)**	89.0% (75.1–102.9)	92.7% (76.4–109.1)	67.9% (45.6–90.2)	85.7% (72.9–98.5)
(b)				
	**ZIKV**			
	**Cook Islands (total)**	**Rarotonga**	**Aitutaki**	**Vanuatu**
**Number of positive ELISA Test results (% of test population)**	176 (65.7%)	141 (67.8%)	35 (58.3%)	102 (51.8%)
**Standardized immunity rates (95% CI)**	64.4% (54.6–74.2)	66.6% (55.3–78.0)	43.9% (27.6–60.2)	58.1% (44.4–71.9)
**Extrapolates immunity rates (95% CI)**	61.0% (49.9–72.2)	63.1% (50.2–75.9)	42.4% (24.0–60.7)	47.4% (36.1–58.6)

**Table A7:** DENV outbreaks in the Cook Islands and Vanuatu until 2016.

Location	Year	DENV Serotype	References
Cook Islands	Prior to 1950	DENV-?	[26]
Cook Islands	1976–1977	DENV-1	[26]
Cook Islands	1980–1981	DENV-4	[26]
Cook Islands	1991–1992	DENV-3	[26]
Cook Islands	1995–1996	DENV-3	[26]
Cook Islands	1997–1998	DENV-2	[26]
Cook Islands	2001–2003	DENV-1	[26]
Cook Islands	2007	DENV-1	[25]
Cook Islands	2008–2009	DENV-4	[25,64]
Cook Islands	2011–2012	DENV-?	[25]
Cook Islands	2014–2015	DENV-1/3	[22]
Vanuatu	Prior to 1950	DENV-?	[26]
Vanuatu	1972	DENV-2	[26]
Vanuatu	1975	DENV-1	[26]
Vanuatu	1979–1980	DENV-4	[26]
Vanuatu	1989	DENV-1	[26]
Vanuatu	1992–1997	DENV-?	[26]
Vanuatu	1998	DENV-2	[26]
Vanuatu	2003	DENV-1	[26]
Vanuatu	2008–2009	DENV-4	[25,64]
Vanuatu	2012–2014	DENV-1	[25,32]
Vanuatu	2013–2014	DENV-3	[25,32]
Vanuatu	2016	DENV-2	[22]

**Table A8:** Time course of DENV/ZIKV/CHIKV outbreaks in French Polynesia, the Cook Islands and Vanuatu during 2012–2016.

Location	DENV Outbreak (Serotype)	ZIKV Outbreak	CHIKV Outbreak	Source
Cook Islands	November 2014–February 2015 (I and III)	February 2014–May 2014	First cases: Oct 2014, local transmission: Jan 2015–Aug 2015	[22,32]
Vanuatu	October 2012–April 2014 (I) December 2013–April 2014 (III)	2014/2015 (exact time unknown)	No reported CHIKV circulation	[22,32,36,37]
French Polynesia	February 2013–May 2016 (possibly up to 2017) (I), February 2013–April 2014 (III)	October 2013–April 2014	Sep 2014–Mar 2015	[16,22,32,44,65,80]
